# Clinical efficacy of immunoglobulin in pediatric patients with severe respiratory syncytial virus pneumonia: a retrospective clinical analysis

**DOI:** 10.3389/fmed.2026.1846957

**Published:** 2026-07-15

**Authors:** Zhiyuan Zhong, Rui Pu, Ran Yao, Xiaoyu Li, Yuqing Ou, Liangkang Lin, Wenqi Yu, Junfeng Xie, Tianqi Liang

**Affiliations:** 1 Department of Pediatrics, Pediatric Hematology Laboratory, Division of Hematology/Oncology, The Seventh Affiliated Hospital of Sun Yat-sen University, Shenzhen, Guangdong, China; 2 Scientific Research Center, The Seventh Affiliated Hospital of Sun Yat-sen University, Shenzhen, Guangdong, China

**Keywords:** children, immunoglobulin, monoclonal antibody, respiratory syncytial virus, severe treatment

## Abstract

**Background:**

The effectiveness of intravenous immunoglobulin (IVIG) in the treatment of severe respiratory syncytial virus pneumonia (SRSVP) remains a topic of ongoing debate. This study aimed to evaluate the association of IVIG on the duration of hospitalization and the use of corticosteroids in pediatric cases of SRSVP.

**Methods:**

To minimize confounding by indication, a 1:1 propensity score matching (PSM) was performed with a caliper width of 0.02. Multivariate logistic regression analysis was conducted for the entire cohort (*n* = 157), while generalized estimating equations (GEE) /generalized linear models (GZLM) were utilized within the matched cohort to evaluate the independent association between IVIG use and clinical outcomes (hospitalization duration and corticosteroid use).

**Results:**

Results showed that intravenous immunoglobulin (IVIG) treatment failed to reduce the duration of corticosteroid use, corticosteroid dosage, or length of hospital stay (OR = 4.634; OR = 4.667; OR = 2.566). Propensity score matching (PSM) successfully generated 41 well-balanced pairs. In the GEE/GZLM analysis (which further corrected for residual baseline imbalances in alanine aminotransferase (ALT), aspartate aminotransferase (AST), and creatinine), IVIG treatment still failed to reduce the duration of glucocorticoid use (OR = 3.229, 95% CI: 1.267–8.232), reduce glucocorticoid dosage (OR = 2.653, 95% CI: 1.063–6.619), or shorten the length of hospital stay (OR = 4.375, 95% CI: 1.581–12.108). Furthermore, subgroup analysis showed that in infants <6 months of age, IVIG treatment did not shorten hospital stay and was not associated with glucocorticoid use outcomes.

**Conclusion:**

In conclusion, intravenous immunoglobulin (IVIG) treatment did not shorten hospital stays or reduce the use of glucocorticoids in children with severe respiratory syncytial virus pneumonia (SRSVP). Therefore, it is necessary to reassess the value of IVIG in the treatment of SRSVP.

## Background

Community-acquired pneumonia (CAP) constitutes a substantial global health burden in children and represents a leading cause of hospitalization and mortality in the pediatric population, particularly among those younger than 5 years of age (1–3). Respiratory syncytial virus (RSV) is widely recognized as the predominant pathogen of CAP in infants and young children worldwide, with nearly all children infected by the age of 3 years (4–6). Importantly, RSV infection in infancy is significantly associated with an increased prevalence and risk of asthma at 5 years of age (relative risk = 1.35), and an estimated 15% of asthma cases at this age could potentially be averted by preventing infant RSV infection (7). Severe RSV infection often requires hospitalization, and critically affected patients may need intensive care unit (ICU) support; in the most severe cases, the infection can be fatal (8). According to a 2019 data analysis, approximately 33 million episodes of RSV-associated acute lower respiratory infection occurred among children aged 0–60 months, leading to 101,400 RSV-related deaths (9). Therefore, early identification and optimal management of severe RSV pneumonia are urgently needed to improve clinical outcomes and reduce long-term respiratory comorbidities in children.

Nevertheless, the efficacy of such interventions remains limited. To date, no specific antiviral agents have been approved for respiratory syncytial virus (RSV) infection, and clinical management is primarily based on symptomatic and supportive care (10, 11). Immunoglobulin, a molecule typically produced by leukocytes during infection, possesses the capability to recognize and bind to viruses, including RSV, thereby facilitating viral clearance. However, evidence regarding the efficacy of intravenous immunoglobulin (IVIG) remains limited and inconclusive. An updated Cochrane systematic review indicated that in children younger than 3 years hospitalized with RSV-associated lower respiratory tract infection, the effect of IVIG on mortality was highly uncertain (RR 0.87, 12). IVIG showed no significant benefit in terms of hospital length of stay, and no notable differences were detected in mechanical ventilation, oxygen therapy requirements, or ICU admission (12). Notably, all included reports of this study were conducted in high-income countries, with a lack of data from high-mortality regions, thereby restricting the generalizability of these results. Furthermore, Moughames et al. suggested that IVIG may shorten ICU length of stay in selected populations, such as immunocompromised patients, especially when administered within 48 h of admission, although it conferred no significant improvement in mortality or readmission rates (13). Nevertheless, this study were not specifically designed for RSV infection and did not assess the efficacy of RSV-specific immunoglobulin. Collectively, current evidence does not support a clear or consistent benefit of IVIG for children with severe RSV pneumonia (SRSVP), highlighting the need for further targeted research. This study aims to investigate whether immunoglobulin exerts clinical effects on the progression of SRSVP through an analysis of clinical data from patients with severe RSV pneumonia.

## Methods

### Study population

This was a retrospective cohort study, including patients hospitalized with respiratory syncytial virus (RSV) infection at The Seventh Affiliated Hospital of Sun Yat-sen University from April 2023 to August 2025. Study data were extracted from the medical records of all patients diagnosed with RSV infection during this period. This study strictly adhered to the principles of the 1964 Helsinki Declaration and its subsequent amendments and was approved by the Medical Ethics Committee of The Seventh Affiliated Hospital of Sun Yat-sen University (Approval No.: KY-2024-312-01). Given the non-interventional and retrospective nature of the study, the Ethics Committee waived the requirement for written informed consent from patients and their legal guardians.

Inclusion criteria were: patients under 18 years of age admitted between April 2023 and August 2025, and diagnosed with severe respiratory syncytial virus pneumonia (SRSVP) according to the “Guideline for the diagnosis, treatment, and prevention of respiratory syncytial virus infection in children in China (2024 Edition)” (14) and the “Guidelines for the management of community-acquired pneumonia in children (2024 revision)” (15).

RSV infection was confirmed by positive respiratory multiplex-PCR or targeted next-generation sequencing (tNGS) using sputum specimens. A positive result for respiratory syncytial virus in either assay was defined as RSV infection.

Exclusion criteria were: age over 18 years, discharge against medical advice, and underlying conditions such as severe malnutrition, chronic heart disease, congenital diseases, malignancy, and multiple trauma. Patients with a history of recurrent respiratory infections, primary immunodeficiency disorders, or secondary immunosuppression were excluded. Immunocompetence was defined clinically based on medical history, with no evidence of recurrent infection, congenital immunodeficiency, or immunosuppressive conditions; routine laboratory testing of serum immunoglobulin levels or lymphocyte subsets was not performed.

### Variables and outcome measurement

To obtain the basic characteristics of eligible patients, we collected patient medical record data from the pediatric inpatient records of The Seventh Affiliated Hospital of Sun Yat-sen University. These characteristics included age, gender, body weight, height, season of onset, history of prematurity, history of wheezing, eczema, allergy history, and presence of siblings, as detailed in Table 1. Grouping variable: Immunoglobulin use: the main exposure variable. Intravenous immunoglobulin (IVIG) was administered as a salvage therapy when wheezing was not relieved after 1–2 days of corticosteroid treatment with informed consent from guardians. The dosage of IVIG was 0.4 g/kg/d, and the treatment course was 3–5 days with a total dose of 1–2 g/kg, adjusted according to clinical remission. Patients were divided into an immunoglobulin group and a non-immunoglobulin group based on this. Corticosteroid therapy was performed with methylprednisolone at an initial dose of 1 mg/kg per administration, and the daily frequency was adjusted based on the degree of wheezing, with a maximum daily dose of 5 mg/kg/d. The drug was tapered and discontinued 1–2 days after wheezing subsided.

**Table 1:** Baseline clinical characteristics of the study population before and after propensity score matching.

Characteristic	Unmatched IVIG (*n* = 60) vs. non-IVIG (*n* = 97)	Unmatched *p*-value	Matched IVIG (*n* = 41) vs. non-IVIG (*n* = 41)	Matched *p*-value
Male, *n*(%)	39 (65.00) vs. 57 (58.76)	0.436	24 (58.50) vs. 29 (70.70)	0.383
Age (years)	0.57 (0.20, 1.28) vs. 0.87 (0.40, 2.30)	0.008	0.60 (0.21, 1.25) vs. 0.69 (0.34, 1.44)	0.257
Body weight (kg)	9.00 (6.05, 11.15) vs. 9.55 (7.50, 12.50)	0.070	9.40 (6.10, 11.10) vs. 9.20 (7.10, 11.50)	0.464
Height (cm)	70.00 (59.25, 77.75) vs. 75.00 (66.00, 88.00)	0.010	72.05 ± 14.46 vs. 73.77 ± 13.80	0.605
BMI (kg/m^2^)	16.91 (15.10, 19.26) vs. 15.97 (15.22, 17.69)	0.072	17.14 ± 2.59 vs. 17.07 ± 2.12	0.895
Co-infection with other pathogens, *n*(%)	56 (93.33) vs. 94 (96.91)	0.429	37 (90.20) vs. 38 (92.70)	1.000
Fever, *n*(%)	38 (63.33) vs. 68 (70.10)	0.379	26 (63.40) vs. 29 (70.70)	0.629
Cough, *n*(%)	59 (98.33) vs. 97 (100.00)	0.382	40 (97.60) vs. 41 (100.00)	1.000
Wheezing, *n*(%)	51 (85.00) vs. 77 (79.38)	0.378	36 (87.80) vs. 35 (85.40)	1.000
Wet rales, *n*(%)	60 (100.00) vs. 97 (100.00)		41 (100.00) vs. 41 (100.00)	
Tracheal tug/subcostal retraction, *n*(%)	39 (65.00) vs. 63 (64.95)	0.995	26 (63.40) vs. 29 (70.70)	0.648
HR (beats/min)	136.00 (130.00, 150.00) vs. 134.00 (126.00, 145.50)	0.410	136.63 ± 15.70 vs. 138.49 ± 13.55	0.527
RR (breaths/min)	42.00 (36.00, 53.75) vs. 40.00 (34.50, 48.00)	0.249	38.00 (34.00, 45.00) vs. 36.00 (32.40, 40.00)	0.246
Initial SpO_2_ (%)	93.00 (90.00, 97.0) vs. 94.00 (91.00, 97.00)	0.500	93.00 (90.00, 97.0) vs. 94.00 (91.50, 98.00)	0.881
Lung consolidation, n (%)	6 (6.19) vs. 3 (5.00)	1.000	2 (4.90) vs. 3 (7.30)	1.000
Bilateral lung involvement, n (%)	71 (73.2) vs. 51 (85.00)	0.084	37 (90.20%) vs. 31 (75.60)	0.180
WBC (×10^9^/L)	8.27 (5.35, 11.15) vs. 7.61 (5.81, 11.31)	0.516	8.61 ± 4.00 vs. 9.18 ± 3.99	0.557
L(%)	47.35 (33.43, 58.95) vs. 54.30 (38.95, 66.90)	0.115	47.40 ± 17.16 vs. 52.75 ± 16.86	0.123
N(%)	40.65 (26.90, 55.68) vs. 34.00 (21.45, 50.10)	0.157	41.75 ± 18.00 vs. 35.93 ± 17.69	0.100
Hb(g/L)	115.00 (106.00, 122.00) vs. 113.00 (104.00, 123.00)	0.600	114.95 ± 12.25 vs. 111.34 ± 10.88	0.134
PLT (×10^9^/L)	335.50 (278.00, 418.75) vs. 328.00 (256.50, 423.50)	0.628	349.44 ± 84.92 vs. 345.02 ± 97.29	0.816
CRP elevation, n (%)	14 (23.33) vs. 27 (27.84)	0.533	9 (22.00) vs. 11 (26.80)	0.791
PCT, n (%)		0.514		0.380
<0.05 ng/mL	41 (42.27) vs. 24 (40.00)		15 (36.60) vs. 19 (46.30)	
(0.05, 2) ng/mL	35 (58.33) vs. 51 (52.58)		22 (61.00) vs. 20 (48.80)	
≥ 2 ng/mL	1 (1.67) vs. 5 (5.15)		1 (2.40) vs. 2 (4.90)	
ALT(U/L)	23.70 (18.75, 33.53) vs. 21.40 (17.04, 31.70)	0.077	25.80 (19.27, 37.14) vs. 21.80 (17.66, 31.45)	0.022
AST(U/L)	41.02 (33.59, 60.63) vs. 41.66 (35.70, 49.95)	0.767	44.90 (32.85, 63.44) vs. 41.70 (35.85, 49.02)	0.088
ALB(g/L)	41.86 ± 3.64 vs. 41.76 ± 4.41	0.874	42.06 ± 4.58 vs. 42.43 ± 3.60	0.669
LDH(U/L)	296.21 (250.85, 329.05) vs. 308.60 (273.39, 344.85)	0.141	296.28 (256.82, 339.10) vs. 326.29 (282.31, 360.08)	0.382
CKMB(U/L)	19.81 (11.88, 29.14) vs. 18.32 (11.96, 26.58)	0.451	20.81 ± 11.32 vs. 20.55 ± 8.27	0.104
Urea (mmol/L)	2.74 ± 0.98 vs. 2.81 ± 1.24	0.677	2.81 ± 1.02 vs. 2.43 ± 1.08	0.099
Creatinine (μmol/L)	22.70 ± 5.46 vs. 24.30 ± 6.24	0.092	22.64 ± 5.78 vs. 21.98 ± 4.91	0.565
Corticosteroid use, n (%)	60 (100.00) vs. 90 (92.78)	0.044	41 (100.00) vs. 40 (97.60)	1.000
Max daily corticosteroid dose (mg/kg/d)	3.00 (2.00, 4.00) vs. 2.00 (1.00, 2.30)	<0.001	3.00 (2.00, 4.00) vs. 2.00 (1.65, 3.00)	0.010
Duration of corticosteroid use (days)	5.00 (4.00, 7.00) vs. 4.00 (3.00, 5.00)	<0.001	5.00 (4.00, 7.00) vs. 4.00 (2.50, 5.00)	0.012
Hospitalization duration (days)	8.00 (7.00, 9.75) vs. 6.00 (5.00, 7.00)	<0.001	8.00 (7.00, 9.00) vs. 6.00 (6.00, 8.00)	<0.001

Propensity scores were calculated using a multivariate logistic regression model including the following baseline variables: age, height, weight, respiratory rate, bilateral lung involvement and baseline SpO_2_. Data are expressed as mean ± standard deviation, median [interquartile range (IQR)], or number (percentage). For unmatched groups, continuous variables were compared using the independent-samples *t*-test or Mann–Whitney U test, and categorical variables were compared using the Chi-square test or Fisher’s exact test. For 1:1 propensity score-matched groups, continuous variables were compared using the paired-samples *t*-test (for normal distribution) or Wilcoxon signed-rank test (for non-normal distribution), and categorical variables were compared using the McNemar test or marginal homogeneity test.

### Statistical analysis

All data were analyzed using IBM SPSS Statistics 25.0 software (IBM Corporation, Armonk, NY, United States). Descriptive statistics were presented as mean ± standard deviation (SD), median (interquartile range, IQR), or counts and percentages, depending on data type. Specifically, continuous variables were expressed as mean ± SD or median (IQR) and compared using paired t-tests or Wilcoxon rank-sum tests; categorical variables were reported as counts and percentages and compared using chi-square tests.

To explore the potential association between IVIG use and clinical outcomes of respiratory syncytial virus (RSV) infection, we constructed multivariate Logistic regression models, calculating odds ratios (OR) and their 95% confidence intervals (CI), with statistical significance set at *p* < 0.05. Given the multicollinearity among age, height, and body weight, we performed two separate regression analyses. 1:1 propensity score matching (PSM) was performed with a caliper width of 0.02, using matching variables including age, gender, body weight, height, respiratory rate at admission, baseline percutaneous oxygen saturation (SpO₂), and bilateral lung involvement.

## Results

**Figure 1:**
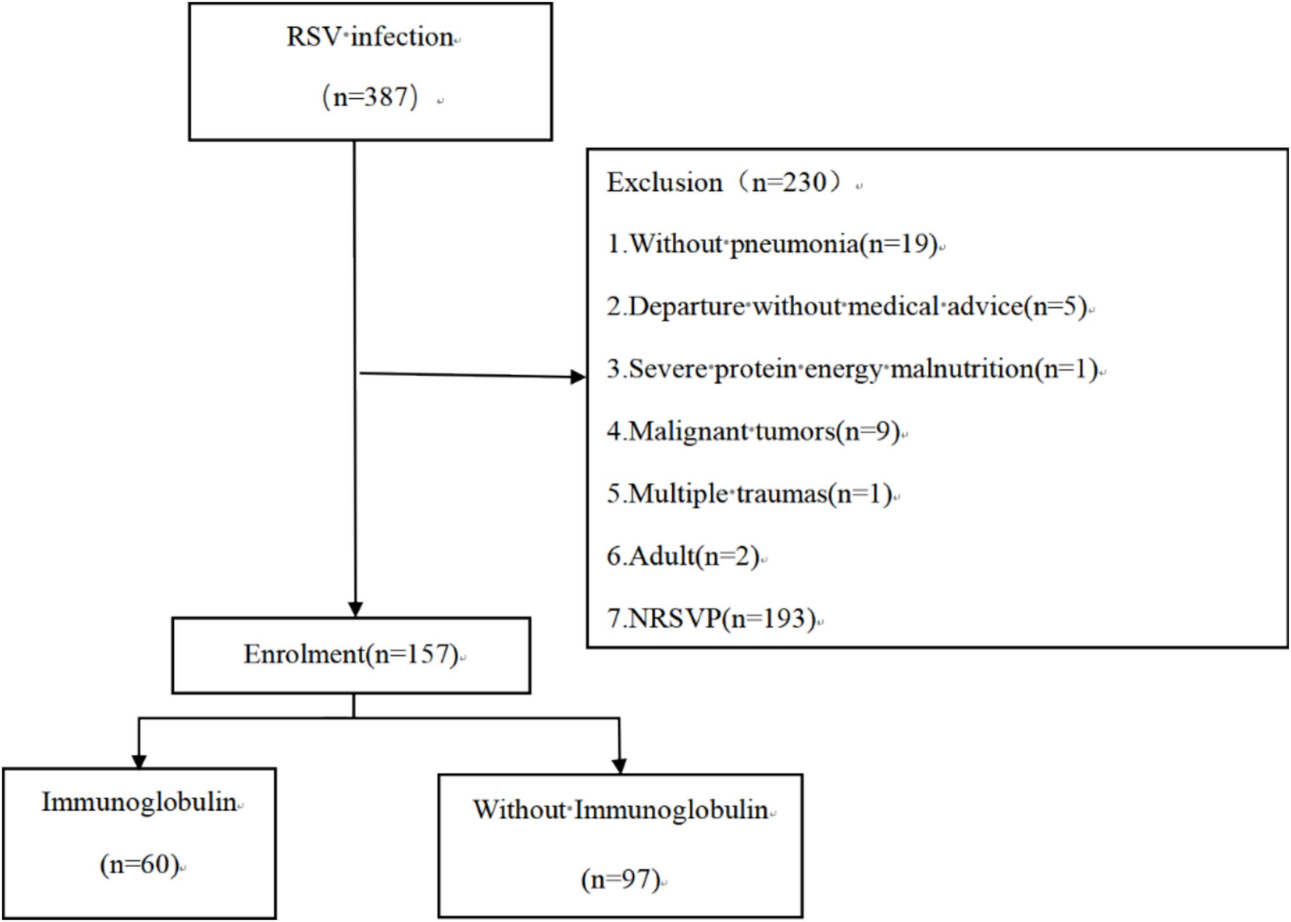
Selection process for severe respiratory syncytial virus pneumonia (SRSVP) patients based on immunoglobulin use.

From April 2023 to August 2025, the Pediatric Medical Center of The Seventh Affiliated Hospital of Sun Yat-sen University admitted a total of 387 patients hospitalized for respiratory syncytial virus infection. Of these, 350 patients met the diagnostic criteria for respiratory syncytial virus pneumonia. During the initial screening process, 230 patients were excluded for not meeting the study’s inclusion criteria. Specific exclusion reasons were as follows: 193 cases were non-severe respiratory syncytial virus pneumonia (NRSVP), 19 patients were diagnosed with upper respiratory tract infection, 5 patients were discharged against medical advice, 1 patient had severe protein malnutrition, 9 patients had malignancy, 1 patient was admitted due to multiple trauma, and 2 patients were adults. A total of 157 (44.9%) patients diagnosed with severe respiratory syncytial virus pneumonia (SRSVP) were finally included in the analysis, of whom 60 received immunoglobulin and 97 did not (see Figure 1).

### General characteristics of the IVIG and non-IVIG groups

In this study, we conducted a detailed analysis of the basic characteristics of a total of 157 patients with SRSVP, divided into two groups. Were initially enrolled. Before propensity score matching (PSM), Results showed that patients in the IVIG group had a median age of 0.5 years, weight of 9 kg, and height of 70 cm. Statistical analysis indicated significant differences (*p* < 0.05) between the two groups in age and height, with the IVIG group having lower a younger age and shorter height than the Non-IVIG group. Furthermore, patients in the IVIG group had significantly faster heart rates and higher respiratory rates, and lower transcutaneous oxygen saturation (SpO_2_) upon admission, suggesting that children patients in the IVIG group may have had more severe illness a higher baseline clinical severity.

Regarding other baseline clinical characteristics, there were no significant differences (*p* > 0.05) between the two groups in body weight, BMI, co-infection with other pathogens, fever, wheezing, inflammatory markers, blood biochemistry, and imaging-reported (radiographic) lung consolidation ratio. This indicates that these factors may not be primary determinants of whether immunoglobulin was used in severe respiratory syncytial virus pneumonia, indicating that these factors were not primary determinants for IVIG administration. After 1:1 propensity score matching, 41 matched pairs were included, and all baseline characteristics were well balanced between groups (all *p* > 0.05). To control for these baseline imbalances and minimize confounding by indication, a 1:1 PSM analysis was performed with a caliper width of 0.02, successfully generating 41 well-matched pairs (41 patients in the IVIG group and 41 in the Non-IVIG group). Following matching, all demographic data and baseline clinical characteristics achieved an optimal balance between the two groups (all *p* > 0.05), as detailed in Table 1.

Importantly, within the propensity score-matched cohort, the clinical outcomes consistently mirrored the trends observed in the unmatched analysis. Compared to the matched Non-IVIG group, the matched IVIG group still. However, the IVIG group had a longer hospitalization duration (8 days vs. 6 days, *p* < 0.01), higher corticosteroid dosage (3 mg/kg/d vs. 2 mg/kg/d, p < 0.01), and longer duration of corticosteroid use (5 days vs. 4 days, p < 0.01) compared to the Non-IVIG group. Detailed data are presented in Table 1.

### Multivariate logistic regression analysis between IVIG and non-IVIG groups

Based on the univariate analysis results, a multivariate logistic regression analysis (for the entire cohort) and generalized estimating equations (GEE) /generalized linear models (GZLM) (for the propensity score-matched cohort) were conducted to investigate whether IVIG use was independently associated with prolonged hospitalization duration, higher corticosteroid dosage, and an extended duration of corticosteroid use in patients with SRSVP. After adjusting for potential confounding factors selected from the univariate analysis (including age, heart rate, respiratory rate, baseline transcutaneous oxygen saturation, and bilateral lung involvement), the multivariate logistic regression model for the entire cohort showed that IVIG treatment was not associated with reduced corticosteroid use, lower corticosteroid dose (OR = 4.634, 95%CI: 2.185–9.827), or shorter hospitalization (OR = 2.566, 95%CI: 1.136–5.799). In the propensity score-matched cohort, the GEE/GZLM analysis—which further adjusted for alanine aminotransferase (ALT), aspartate aminotransferase (AST), and creatinine derived from the univariate analysis—yielded highly consistent results, confirming that IVIG treatment did not confer beneficial effects on these clinical outcomes (Table 2). Subgroup analysis was further conducted for infants aged <6 months using the GEE/GZLM approach with the same covariates adjusted. The results indicated that in this low-age subgroup, IVIG treatment still did not reduce the risk of prolonged hospitalization (>7 days) (OR = 4.375, 95%CI: 1.581–12.108), whereas no statistically significant association was observed for corticosteroid usage parameters. Detailed data are presented in Tables 2, 3.

**Table 2:** Impact of IVIG use on corticosteroid use and hospitalization duration.

Characteristic	Unmatched cohort (*n* = 157)		Matched cohort (*n* = 82)	
	Adjusted OR (95% CI)	Significance	Adjusted OR (95% CI)	Significance
Corticosteroid use ≥5 days	4.634 (2.185–9.827)	<0.001**	3.229 (1.267–8.232)	0.014*
Corticosteroid dose >2 mg/kg/d	4.667 (2.182–9.980)	<0.001**	2.653 (1.063–6.619)	0.037
Hospitalization duration >7 days	2.566 (1.136–5.799)	0.023*	4.375 (1.581–12.108)	0.004**

Unmatched Cohort: Adjusted for: Age, heart rate, respiratory rate, oxygen saturation, bilateral lung involvement; Matched Cohort: Adjusted for: ALT, AST, Urea. *p < 0.05, **p < 0.05.

**Table 3:** Impact of IVIG use on corticosteroid use and hospitalization duration in young infants (age ≤ 6 months).

Characteristic	Unmatched cohort (*n* = 58)		Matched cohort (*n* = 32)	
	Adjusted OR (95% CI)	Significance	Adjusted OR (95% CI)	Significance
Corticosteroid use ≥5 days	1.375 (0.419–4.509)	0.599	3.279 (0.462–23.253)	0.235
Corticosteroid dose >2 mg/kg/d	3.396 (0.929–12.420)	0.065	2.887 (0.518–16.095)	0.226
Hospitalization duration >7 days	2.319 (0.676–7.950)	0.181	5.644 (1.044–30.506)	0.044*

Unmatched Cohort: Adjusted variables: Wheezing, tracheal tug/subcostal retraction, days of oxygen therapy, lymphocyte count. Matched Cohort: Adjusted for: ALT, AST, Urea. *p < 0.05.

## Discussion

This study conducted an in-depth analysis of pediatric patients hospitalized with RSV infection between 2023 and 2025. Our findings reveal that, in clinical practice, children receiving intravenous immunoglobulin (IVIG) treatment typically presented with more severe illness, primarily evidenced by higher heart rates, respiratory rates, and lower oxygen saturation levels upon admission. However, multivariate logistic regression analysis, after adjusting for confounding factors such as disease severity, indicated that IVIG use did not associated with improved outcomes. On the contrary, IVIG was not associated with shorter hospitalization durations or lower intensity corticosteroid use (OR = 2.57; OR = 4.67). Notably, these associations remained statistically significant after 1:1 propensity score matching. Importantly, this persistent association should not be interpreted as a causal effect of IVIG, but rather indicates that residual confounding by indication was not fully eliminated even after PSM. In clinical practice, IVIG was still preferentially administered as a salvage therapy to infants with more severe baseline conditions, which continued to confound the observed relationship between IVIG and clinical outcomes. This suggests that despite IVIG often being employed as a salvage therapy for severe respiratory syncytial virus pneumonia (SRSVP), its benefits in shortening the disease course or impeding progression are very limited. This aligns with previous research conclusions, indicating that once the disease progresses to a severe stage, existing anti-inflammatory and non-specific immunomodulatory interventions often struggle to reverse established pathological damage (16, 17). The subgroup analysis further showed that IVIG use did not improve clinical outcomes in infants aged <6 months. This might be attributed to the influence of maternally transmitted antibodies, where infants in this age group may still retain certain levels of maternal RSV-neutralizing antibodies, and the addition of exogenous non-specific IVIG could result in immune dilution or interference. Alternatively, it could be due to the limited responsiveness of the immature immune system of young infants to non-specific immunoglobulins (18–20). This result suggests the necessity of shifting the focus of prevention and control to the early prophylactic stage before infection occurs.

Our findings are highly consistent with high-level international evidence and landmark clinical trials. In a major, large-scale multicenter randomized controlled trial (RCT) conducted by Rodriguez et al., the therapeutic administration of respiratory syncytial virus immune globulin intravenous (RSV-IGIV) in hospitalized children failed to significantly reduce the duration of hospitalization, intensive care unit stay, or the requirement for mechanical ventilation compared to the placebo group (21). Furthermore, a comprehensive Cochrane systematic review synthesizing multiple international RCTs formally concluded that neither standard intravenous immunoglobulin (IVIG) nor pathogen-specific immunoglobulins offer a distinct clinical benefit in accelerating recovery or shortening the clinical course of severe RSV lower respiratory tract infections (12). While some early, unadjusted retrospective studies historically suggested a vague immunomodulatory benefit of IVIG, these correlations were often confounded by baseline selection bias and institutional prescribing variations. By implementing a robust 1:1 PSM framework coupled with GEE/GZLM doubly robust adjustments to minimize these selection artifacts, our real-world data strongly align with these high-quality experimental paradigms, demonstrating that routine, unselected IVIG utilization does not yield pragmatic clinical efficacy in severe pediatric RSV pneumonia.

With ongoing advancements in RSV F protein structure research, RSV control is undergoing a paradigm shift from treatment to prevention. Monoclonal antibodies (mAbs), with their high pathogen specificity, are emerging as “magic bullets” against infections. For instance, Nirsevimab targets a highly neutralization-sensitive epitope on the RSV pre-F protein and utilizes Fc mutation technology to extend its half-life, achieving a breakthrough where a single injection can protect infants throughout the entire RSV epidemic season (22, 23). Studies have shown that Nirsevimab offers a protective efficacy of 70–80% against RSV-associated lower respiratory tract infection (LRTI) in both preterm and full-term infants (24–26). In contrast, traditional Palivizumab, while reducing hospitalization rates by 55%, has very limited population coverage due to the need for monthly administration and its restriction to high-risk infants (27, 28). Newer long-acting monoclonal antibodies (such as Clesrovimab) have demonstrated efficacy in preventing severe RSV that is comparable to or even superior to traditional regimens (29). This further supports the argument for establishing a high-level immune barrier before infection occurs, rather than passively neutralizing the virus after widespread dissemination.

Children, as the primary target population for RSV prevention, are witnessing a historic shift in control strategies from passive response to proactive planning. By 2023, the US FDA approved two landmark new preventive products: Nirsevimab (a long-acting mAb for infants) and an RSV vaccine for pregnant women, both recommended by the CDC’s Advisory Committee on Immunization Practices, signaling RSV prevention entering an era of population-wide protection (30).

Tailored prevention strategies for the pediatric population are becoming increasingly well-defined: passive immunoprophylaxis with long-acting monoclonal antibodies (such as Nirsevimab) for infants under 6 months; the exploration of active immunization via live-attenuated vaccines for older infants over 6 months; and maternal vaccination during late gestation to induce transplacental antibody transfer for neonatal protection (31). The advantage of this layered strategy is that it builds a continuous protective barrier from prenatal to infancy, addressing the immune characteristics and exposure risks of different age groups.

This study has several limitations that warrant consideration. First, as a single-center study, the sample size of patients with SRSVP (*n* = 157) restricted the statistical power of certain subgroup analyses, particularly when analyzing interactive effects within infants aged <6 months. Second, although both multivariate logistic regression and propensity score matching (PSM) combined with generalized estimating equations (GEE) /generalized linear models (GZLM) were used to adjust for observed confounding factors, the retrospective design prevents the complete elimination of “confounding by indication.” Because IVIG was preferentially administered to infants with higher baseline clinical severity, these findings may partially reflect a more aggressive underlying disease course rather than a direct textbook causal relationship. Additionally, our statistical models could not completely exclude the influence of unmeasured factors such as viral load, baseline immunological status (including serum immunoglobulin levels), genetic susceptibility, or the precise timing of IVIG or corticosteroid administration (early vs. late in the disease course). Future prospective, multi-center, randomized controlled trials should further explore and validate these associations by implementing standardized immunomodulatory protocols and tracking long-term clinical outcomes.

In summary, this study indicates that in the treatment of severe RSV pneumonia, IVIG, despite often being used as a salvage measure, did not demonstrate clear benefits in shortening the disease course or improving prognosis, and it carries significant economic costs. Clinicians should carefully evaluate its indications for use. Given the current limitations of specific treatment options, future efforts should actively promote early prevention strategies centered on long-acting monoclonal antibodies and vaccination, fundamentally transforming the RSV prevention and control paradigm from severe disease treatment to early interception.

## Data Availability

The datasets presented in this article are not readily available because: none. Requests to access the datasets should be directed to zhongzhiyuan@sysush.com.
